# From policy to practice: An assessment of biosecurity practices in cattle, sheep and goats production, marketing and slaughter in Baringo County, Kenya

**DOI:** 10.1371/journal.pone.0266449

**Published:** 2022-04-07

**Authors:** Edna N. Mutua, Bernard K. Bett, Salome A. Bukachi, Benson A. Estambale, Isaac K. Nyamongo

**Affiliations:** 1 Institute of Anthropology, Gender and African Studies, University of Nairobi, Nairobi, Kenya; 2 Animal and Human Health Group, International Livestock Research Institute, Nairobi, Kenya; 3 Research, Innovation and Outreach, Jaramogi Oginga Odinga University of Science and Technology, Bondo, Kenya; 4 Cooperative Development, Research and Innovation, The Cooperative University of Kenya, Nairobi, Kenya; Bangladesh Agricultural University, BANGLADESH

## Abstract

Globally, biosecurity is instrumental in prevention, control and management of livestock diseases and protection of human health. It is defined, prescribed, adopted and enforced through global, regional and national frameworks, laws, policies and strategies. There is more biosecurity practice research conducted in developed countries than developing ones. Consequently, the gap between the ideals recommended in biosecurity frameworks and what is practical in under-resourced rural settings is poorly understood. This anthropological study sought to assess adoption of biosecurity practices across a cattle, sheep and goat value chains continuum to demonstrate where risks lie. The cross-sectional mixed-methods study took place in Baringo County, Kenya. Qualitatively, it utilized 26 focus group discussions with community members and 10 observational interviews with slaughter facility workers. Quantitatively, it included a household survey with 560 community members and a separate survey with 231 livestock traders. Results show that producers, traders and slaughter facility workers did observe some biosecurity practices but not others due but not limited to personal preference, limitations in veterinary service delivery and enforcement of some biosecurity measures, and lack of requisite infrastructure. The study concludes that the implementation of biosecurity measures in rural settings is more complex than envisioned in biosecurity policies and frameworks. It can be hampered by resource limitations, poor enforcement, and contestations with cultural practices. The study recommends that further studies on willingness to adopt biosecurity measures targeting community members in under-resourced settings be conducted to identify possible critical points of intervention at county and national levels.

## Introduction

Biosecurity plays an important role in livestock disease prevention, control and management at global and national levels. It is defined by the Food and Agriculture Organization (FAO) as “a strategic and integrated approach to analysing and managing relevant risks to human, animal and plant life and health and associated risks to the environment (FAO 2007; page 96)” [1]. With the increase in global and local movement of people and livestock; trade in livestock and livestock products; spread of livestock and plant diseases and pests; spread of invasive alien species; and development and use of genetically modified organisms, it has become important for governments to take biosecurity measures to protect their territories, citizens, livestock, plant life and environment from associated risks.

Adoption and enforcement of biosecurity measures is achieved through global, regional and national regulatory frameworks and a multiplicity of application and enforcements activities by various allied actors. Internationally, an example of a biosecurity regulatory framework is the Codex Alimentarius whose focus is food safety and animal health [1]. By becoming signatories to this framework, countries agree to abide by high standards of food safety and animal health. This does not however limit individual countries from developing their own biosecurity regulations [1]. Besides being a signatory of the above international biosecurity framework, Kenya is also party to regional biosecurity standards under the East Africa Community [2]. An example are the East African Community quality standards on raw milk, yoghurt, whole milk and skimmed milk powder, pasteurized milk, sweetened condensed milk, ultra-heat treated milk and dairy ices and ice-creams [2].

As a nation, Kenya also has its own local biosecurity frameworks in form of Food safety laws, policies and strategies [3]. Examples of food safety laws focused on production of livestock and allied products, marketing and slaughter are the Meat Act, the Animal Diseases Act, the Dairy Act and the Dairy Industry Regulations [3]. The Meat Act provides guidelines on the powers of the director of veterinary services; categorization of slaughter houses; construction and upgrading of slaughter facilities; meat inspection; disposal and destruction of animals or meat; and hygiene for workers, slaughter facilities and equipment, and animal products [4]. The Dairy Industry Act mandates the Kenya Dairy Board to set national standards of production, transportation, storage, packaging and trade of dairy produce and conduct inspections to ensure that they are adhered to [5]. In addition, dairy industry regulations spell out responsibilities to individual and institutional producers, traders, and manufacturers of milk and milk products on the aspects of hygiene, packaging, labelling, transportation [6]. Regulations concerning the movement of animals locally and internationally for any purpose, such as the need for permits when moving animals from one place to another, hygiene standards to be observed when moving livestock in vehicles, and the requirement to observe disease containment measures set by veterinary departments when notifiable diseases occur, are outlined in the Animal Diseases Act [7]. Relevant policies and strategies include but are not limited to the national food and nutrition security policy 2011, the national food safety policy 2013, the Kenya veterinary policy 2015, the communication strategy on prevention and containment of antimicrobial resistance 2018–2020 and the economic recovery strategy for wealth and employment creation 2003–2007 [3].

While there are biosecurity guidelines at the international, regional and national levels, the gap between observation of the ideal requirements and what is practical locally, particularly in under resourced rural settings, is not clearly understood. Biosecurity practice research is more common in developed countries than the developing ones [8], hence the limited understanding of its uptake in the latter [9]. The research has also been more focused on producers than other actors in the value chains considered for study [10]. This anthropological study sought to assess adoption of biosecurity practices across a cattle, sheep and goat value chains continuum to demonstrate where risks lie, identify possible critical points of intervention and to inform biosecurity policy at county and national levels.

## Methods

### Study area

**Fig 1:**
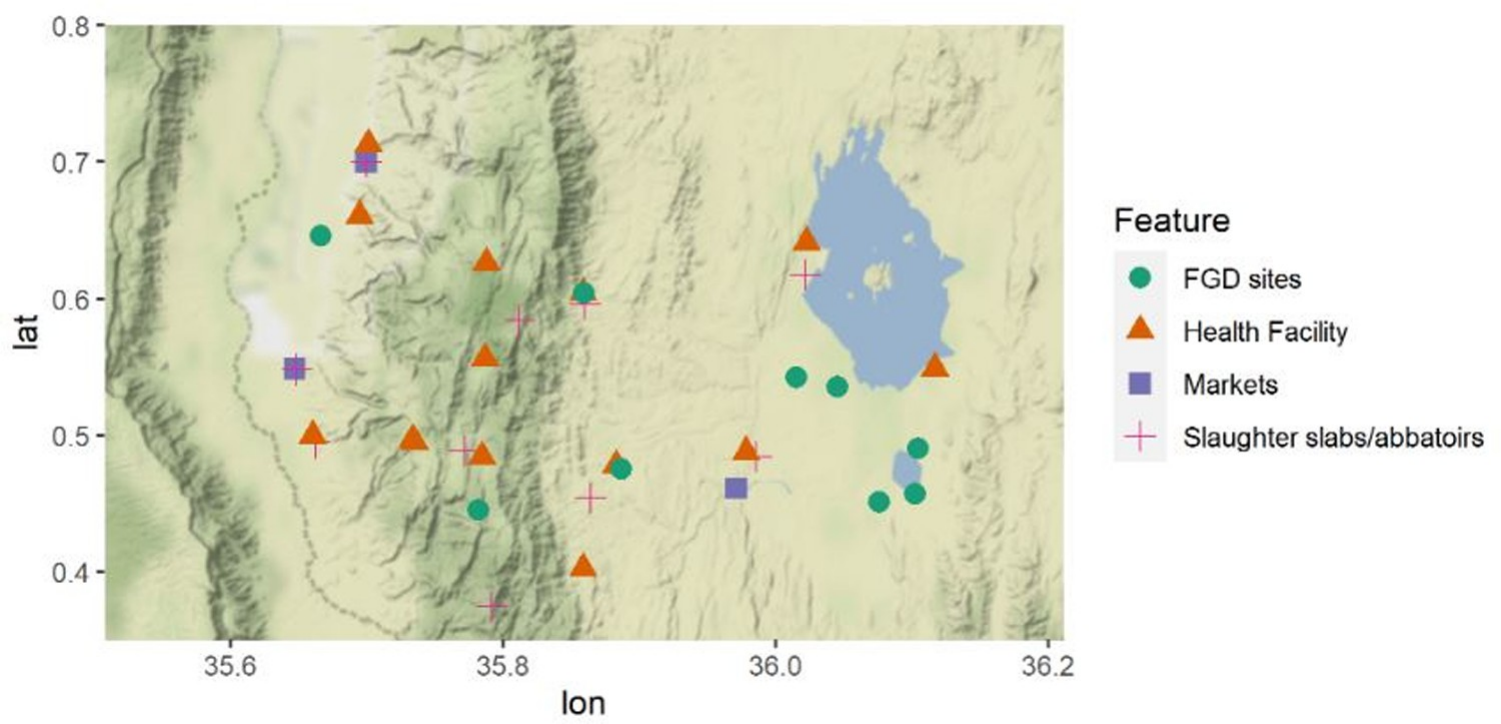
Map of study site.

The study took place in the Central, North and Marigat sub-counties of Baringo County from 2015–2016. Altitude ranges were used to segment the study site into four zones namely; the highland, midland, lowland and riverine zones (Fig 1). The highland, midland and riverine zones were predominantly inhabited by Tugen speakers, a subtribe of the Kalenjin community, who are agro-pastoralists. The lowland zone was predominantly inhabited by the Ilchamus, a subtribe of the Maasai community, who were sedentary pastoralists. The County’s main economic activity was livestock farming. In 2015, Baringo County was estimated to have 3.47%, 2.23% and 2.08% of Kenya’s goats, cattle and sheep (MoLD 2015). The three main markets within the study site were Kapatara and Barwessa in the riverine zone and Marigat in the lowland zone.

### Study design, sampling and data collection

The study applied a cross-sectional research design utilizing both qualitative and quantitative techniques. At the producer level, biosecurity data was collected through a knowledge, attitudes and practices survey. The survey covered knowledge of transmission pathways for zoonotic disease (Rift Valley Fever), patterns of livestock products handling and consumption, livestock disease management, disposal of dead stock and grazing patterns. The survey tool was pretested among 40 participants whose data were not part of the main sample. Survey respondents were selected through systematic sampling from 20 clusters, 5 per zone, formed to facilitate fair distribution within the zones. The survey sample was determined using the proportion to size sampling methodology for a finite population which resulted in 383 respondents. An additional 5% was included to cover for possible incomplete questionnaires resulting in a sample of 400. In an aim to increase the external validity of the survey findings, the sample size was increased by 8 respondents per cluster resulting to 560 respondents.

Twenty-six focus group discussions (FGDs) involving 231 discussants were conducted with purposively selected community members. Of these, half were men only discussions and the other half were for women only. To participate, community members had to be current livestock keepers and be knowledgeable of common livestock diseases affecting cattle, sheep and goats in their locality and farmer practices in consumption patterns of livestock products, livestock disease management and grazing/watering patterns.

Data on biosecurity practices in livestock trade was collected in November 2015 from, Marigat, Barwessa and Kaptara, the main live animal markets within the study site in which cattle, goats and sheep are traded. A total of 231 traders were interviewed. In each market, interviews were carried out on two market days, targeting new traders through convenience sampling. Traders who frequented more than one of the three markets were only interviewed in the market of first contact. Interviews focused on the frequency of operations, sources of livestock for trade, uses of purchased stock, disease control and disposal of animals that die in transit.

At the slaughter facilities level, biosecurity data was collected through ten interviews with personnel, which included observation of operations in 3 abattoirs and 7 slaughter slabs sampled purposively. Personnel were interviewed on the daily operations of the slaughter facilities which included frequency of slaughtering, livestock volumes processed per week, workforce and disease control practices. Observations were made on the use of protective clothing, disposal of condemned parts and carcasses, disposal of other waste comprising of blood, used water and manure, and accessibility of slaughter areas to rodents, birds and dogs.

### Data management and analysis

Producer data was entered and cleaned in CSPRO version 6.1 and exported to SPSS version 23 for analysis using descriptive statistics and R version 4.0.4 for regression analysis of exposure to risk factors. Among producers, levels of exposure to risk were determined through 22 five-point Likert scale questions with responses ranging from never engaging in a risk practice to always engaging is a risk practice. The questions covered community practices in handling and consumption of meat, milk, and blood; handling and treatment of sick and aborting livestock; and handling of dead livestock and abortus. Each of the 22 responses were recoded 1 if it was a good practice and 0 if not. The binary categorizations were then summed up to get the total number of good practices per respondent. This total sum was then used as the dependent variable to run an ordinary least squares regression model. The independent variables used in the model were zone, sex, marital status, household type, age, education level, ethnicity, and main livelihood activity.

The expression of the linear regression model is given by:

$$\hat{Y}=b_{0}+b_{1}X_{1}+b_{2}X_{2}+\cdots +b_{p}X_{p}$$

Where $\hat{Y}$ is the expected value of the number of good practices, *X*_1_ through *X*_*p*_ are the independent variables representing socioeconomic characteristics while *b*_1_ through *b*_*p*_ are coefficients to be estimated.

The model’s goodness of fit was assessed using the *F*-test, which gives the overall significance of the model. F-statistic was 12.24 and produced a p-value less than <0.001, which is highly significant. This shows that none of the included predictor variables has a zero coefficient. In addition, residuals plots were used to check for regression assumptions.

Livestock trader data was entered, cleaned and analyzed using descriptive statistics in SPSS 23. Observational and interview slaughter facilities data was entered into a spreadsheet and analyzed through content analysis. Focus Group Discussion data was captured through field notes and audio files. The audio files were translated and transcribed into English for analysis by scribes competent in English, Kiswahili and Tugen or IIchamus. Transcribed data was managed and coded into emergent themes in Nvivo 10 (QSR International Melbourne). The emergent themes included, livestock product handling and consumption patterns, handling of sick and aborting livestock, livestock disease treatment, disposal of dead livestock, and grazing and watering patterns. Data on common livestock diseases collected through focus group discussions was compiled into a table detailing the disease English and local names and the zone where they were reported to occur.

### Ethical considerations

Requisite ethical approvals were acquired from the World Health Organization (WHO) and the Kenyatta National Hospital/University of Nairobi Ethics Committees referenced Protocol ID B20278 and P70/02/2013, respectively. Prior to engaging in any research activity, all literate participants were required to give written consent whereas the illiterate consented verbally and provided a thumb print as proof. Only participants of consenting age were allowed to participate in the study.

## Results

### 1. Producer level

#### Producer characteristics

At the producer level, a total of 560 respondents took part in the household survey. Of these, 47.5% were male and 52.5% female. Most of the respondents had primary level education, (52%) and practiced Christianity (99%). Their average age was 44 years, but most were within the 27–35 years range (26.3%). Their primary sources of income were crop (n = 266, 47.5%) and livestock farming (n = 113, 20.2%). A total of 112 men and 119 women aged between 18–84 years with an average age of 41.7 years participated in the focus group discussions (FGDs).

#### Common Livestock diseases in cattle, sheep and goats

In FGDs, diseases were reported through a description of symptoms or direct naming using English or local names (Table 1). English names were commonly mentioned for the diseases that farmers had received livestock vaccines or treatment services for from the local veterinary departments. These diseases include Lumpy Skin Disease (LSD), Foot and Mouth Disease (FMD) and East Coast Fever (ECF). Community members free-listed ECF, FMD, Contagious Bovine Pleuro-Pneumonia (CBPP), LSD, anthrax, trypanosomiasis, brucellosis, foot rot, red water, black quarter, mange and mastitis as the most common cattle diseases in the area. Among sheep and goats, Contagious Caprine Pleuro-Pneumonia (CCPP), ECF, brucellosis, cerebral coenurosis, diarrhea, peste des petite ruminants (PPR), FMD, mastitis, mange, sheep and goat pox and foot rot were reported as most common. Of these diseases, brucellosis and anthrax are zoonotic. Another emerging zoonotic disease, Rift Valley Fever (RVF), was reported in Baringo County for the first time in 2007 and had since not recurred.

**Table 1:** Occurrence of common livestock diseases.

	Livestock species	Livestock diseases (English name)	Livestock disease (local name(s))	Occurrence in zones			
				Highland	Midland	Riverine	Lowland
1	Cattle	East Coast Fever	Cheptigon /esse /malaria	✓	✓	✓	✓
		Contagious Bovine Pleuro-pneumonia	Chebwon	✓	✓	✓	✓
		Foot and mouth disease	Ngorionte / ngorion/ moigoitie/ moigutie	✓	✓	✓	✓
		Lumpy skin disease	✓	✓	✓	✓	✓
		Anthrax	Kiptongok	✓	✓	✓	✓
		Mastitis	✓	✓	✓	✓	✓
		Skin disease that causes the hide’s hair to come off (Mange)	Simbirion/simbiriondet	✓	**-**	✓	✓
		Skin disease	Kiporom	**-**	✓	✓	✓
		Brucellosis	Riobon/regon	**-**	**-**	✓	✓
		Black quarter	✓	**-**	✓	**-**	**-**
		Animal urinates blood (Red water)	Kisiboen/siboen	**-**	✓	✓	**-**
		Trypanosomiasis	Kiplis	**-**	**-**	✓	✓
		Diarrhea	✓	**-**	**-**	✓	**-**
		‘Arthritis’	Chemugui/ cherabony	**-**	**-**	✓	**-**
		Foot rot	✓	**-**	**-**	✓	**-**
2	Sheep	Contagious Caprine Pleuro-pneumonia	Chebwon/chebwone	✓	✓	✓	✓
		Bloating when they eat green grass that is wet with dew	Kiptungus/cheptungus	✓	✓	✓	**-**
		Skin disease (Mange) that causes hair to fall off	Simbirion/ chepsulei	✓	**-**	✓	✓
		Skin disease (Sheep and goat pox)	Kiporom	**-**	**-**	✓	✓
		East Coast Fever	Cheptigon/ esse	✓	✓	**-**	**-**
		Foot rot	✓	✓	**-**	✓	✓
		Intestinal Worms	✓	**-**	✓	✓	✓
		‘Arthritis’—the one which causes swelling at the joints	Cherabony	**-**	✓	**-**	**-**
		Miscarriages when they are 2–3 months pregnant. (these animals are suspected to consume poisonous plants)	✓	**-**	✓	**-**	**-**
		Cerebral coenurosis (an animal circles and has a watery brain upon slaughter)	Kibeiwa/ chebirbirmet/ kibirbirmet	**-**	**-**	✓	**-**
		Diarrhea	✓	**-**	**-**	**-**	✓
		Peste des Petite Ruminants	✓	**-**	**-**	**-**	✓
3	Goats	Contagious Caprine Pleuro-pneumonia	✓	✓	✓	✓	✓
		Mange, a skin disease that makes goats to shed their hair, feel itchy, and develop rashes	Simbirion/ simbiriondet	✓	✓	✓	✓
		Skin disease (Sheep and goat pox) that looks like chickenpox. An animal has many pimples that produce pus. When animals give birth there will be many wounds in the eyes of the newborn which will blind the animal.	Kiporom	**-**	✓	✓	**-**
		East Coast Fever	Cheptigon/ esse	✓	✓	**-**	✓
		Foot and mouth disease	✓	✓	✓	**-**	✓
		Cerebral coenurosis (an animal circles and has a watery brain upon slaughter)	Kibeiwa/ chebirbirmet/ kibirbirmet	**-**	✓	✓	✓
		Bloating	Kiptungus	**-**	✓	**-**	**-**
		Mastitis	✓	**-**	**-**	✓	**-**
		Foot rot (Legs swell from the bottom, produce pus and animals have poor feeding patterns)	Kiborok	✓	✓	✓	✓
		Diarrhea	✓	✓	**-**	✓	✓
		Intestinal worms	✓	**-**	**-**	✓	**-**
		Miscarriages	✓	**-**	**-**	**-**	✓

### Risk at producer level

#### i. Probability of producer infection through food handling and consumption

Milk was frequently boiled before consumption (95.3%, CI 93.9–97), but 2.3% (CI 0.3–4.5) of respondents only boiled milk sometimes and a further 2.3% (CI 0.3–4.5) never did. Nearly all respondents (95.3%, CI 93.9–97) reported to have milked sick animals but only 37.5% (CI 33.6–41.9) reported consuming milk from sick animals. Blood was often consumed raw or cooked in 24.2% (CI 20–28.8) and 43.9% (CI 39.6–48.4) of cases, respectively. When animals were slaughtered at home, 48.7% of respondents (CI 44.4–53.1) never called a veterinary officer or a local expert (51.7%, CI 47.5–56.1) to inspect the meat before consumption. Liver, kidneys and the small intestines were sometimes consumed raw. 27.8% (CI 23.5–32.4) of respondents frequently slaughtered sick animals for consumption whereas 50.8% (CI 46.7–55.3) have consumed meat from a dead animal before.

Focus group discussion data corroborated the survey findings. There was agreement that community members consumed blood in various ways as shown in the following quotes. The blood was sourced through slaughter or extraction from the jugular vein in cattle.

*“People wait and when it comes out [blood] they stir and pour in sufuria (saucepan) to coagulate and look like liver, and remove the serum and mix with the milk, the one in the calabash. And that which has coagulated, they cut and eat that way without cooking.” Female discussant, Highland Zone*.

*“When the animal is cut on the neck you collect the blood and drink when it is warm, others cook, they fry.” Female discussant, Riverine zone*.

*“When we slaughter cattle*, *we put the blood in a container and men eat the blood just like that when it is raw*. *Sometimes*, *we also use an arrow on the neck of cattle*. *We remove the blood from the vein*, *and we mix [it] with milk and take as food*.*” Male discussant*, *Lowland zone*.

Livestock products were not only used for food purposes. They were used as sources of medicine or means of administering traditional forms of treatment as demonstrated below in the case of anemia, poisoning, snake bites or other sickness.

*“Whenever we take children to hospital and the doctors realize that their blood is low [anemia], we come and slaughter a goat and give them some blood mixed with liver.” Female discussant, Lowland zone*.

“If a child eats this (pointing at a poisonous plant) or just any poison, that [animal fat] can treat. You eat the fat from the tail of the sheep.” Male discussant, Midland zone.

“When someone is bitten by a snake you apply [animal fat] on the body [the bite site] and also give to drink.” Female discussant, Midland zone.

“Eyande”, its water is like medicine. It is removed from the stomach of the goat. When you slaughter you just squeeze out [the stomach to collect].” Male discussant, Riverine zone.

Consumption of meat from dead animals was also reported during normal times and the RVF outbreak in 2007 as shown in the following quotes:

*“There are those who say they don’t eat meat from dead animals. So, if you don’t eat someone else says give me that [dead animal] I slaughter. You find people eating.” Male discussant, Riverine zone*.

*“During that RVF outbreak time, despite the fact that people were told [by the veterinary department] not to consume, when animals died, we just continued consuming.” Male discussant, Lowland zone*.

#### ii. Probability of producer infection through animal handling without biosecurity measures

Nearly 9 in 10 respondents (85.3%, CI 82.6–88.1) handled sick animals with bare hands while 8 in 10 (84%, CI 81.3–87) assisted animals with delivery using bare hands. Sick animals and those that aborted were either treated using biomedical or herbal medicines. Most producers (61.6%, CI 57.6–65.8) preferred to buy veterinary medicines and treat the animals by themselves without the guidance of a veterinary officer due to lack of access to one or avoidance of costs associated with acquiring professional services. Disposal of animals was done through multiple ways which included consuming, burying whole, skinning then burying, skinning then giving to dogs, skinning then throwing in the open, burying whole or skinning then burying. Consumption was done as a means of providing food as well as reducing the losses incurred from an animal’s death. Among survey respondents, 40.5% (CI 36.1–45) frequently buried aborted foetuses while 47.5% (CI 43.1–51.9) gave them to dogs for consumption and 13.6% (CI 9.6–17.8) threw them in the open to rot. Further 40.5% (CI 36.2–44.9) frequently buried, 14.2% (CI 9.8–18.7) burnt cadavers from sick animals.

Focus group discussion data agreed with the survey finding on non-use of protective gear when handling sick animals or assisting animals with delivery. The idea of using gloves was new to them as demonstrated in the quotes below. The discussants also cited the challenges with deliveries in that they could occur away from places with ample access to protective gear and water for hand washing.

*“It is very funny, using gloves on a cow also?” Female discussant, Highland zone*.

*“That practice has come recently. Even when we help them give birth, we don’t wear gloves. Normally, we help each that way.” Female discussant, Lowland zone*.

*“Again*, *in case a cow or goat gives birth in the bush and there is no water*, *you can use soil to dry your hands but if is at home we just use wate*r.” *Male discussant*, *Lowland zone*.

Skinning of dead animals as reported by a female discussant from the highland zone, was also driven by the belief that, *“when an animal is buried with the skin*, *it causes harm to the remaining stock*, *they might die”*, besides the need for food. Livestock treatment was mainly done using conventional and herbal medicines by the farmers themselves. The farmers purchase conventional medicines and store them at home for use when needed, as written on the instruction labels or as they have learnt to use from other people as shown below:

*“It was there, “chebwon” [CBPP], and affected goats and others. This is too much. We now buy medicine. We stay with the medicine and they [medicines] take care of them [animals].” Male discussant, Midland zone*.

*“We just learnt from the old men. When a veterinary officer is there, we just observe what they do then we continue to inject [like they did].” Male discussant, Riverine zone*.

*“We estimate [the quantity of medicine necessary to effect treatment]. We found people doing that and we continued the practice.” Female discussant, Riverine zone*.

Some traditional medicines/herbs, usually sourced locally and used in treatment of cattle, sheep and goats included “*soget”*, *Warburgia ugandensis*, used in the highland and riverine zones to treat *“chebwon”* (CCPP) and to treat ECF in the midland zone. In addition, “*koloswo”*, *Terminalia kilimandscharica*, was used to treat “*chebwon”* in the riverine zone. *“Yemit”*, *Olea europaea*, was used to treat *“cheptigon”*, ECF, in the midland zone. In the lowland, *“sukuroi”*, *Aloe sp*. were used in treatment when “goats were coughing.”

#### iii. Probability that animals get infected with disease through grazing and watering

Mature livestock were mainly grazed and watered away from homesteads except in zero grazing systems adopted by few farmers in the highland zone. Therefore, 80.8% (CI 77.8–84.1) of 541 respondents reported that their livestock frequently came into contact with other animals when grazing locally whereas 85.2% (CI 82.6–88.1) added that their animals were watered in the same water points as other animals. Nearly 7 in 10 (66.2%, CI 62.3–70.1) reported that their animals frequently grazed outside the locality, particularly during the dry season which lasted from January-April but sometimes extended to June. As reported in focus group discussions, producers associated mixing of animals from different homesteads with spread of diseases, parasites like ticks, inbreeding and crossbreeding with low quality animals as reflected in the quotes below:

*“They can have ticks and transfer to other animals, also cross breeding with poor quality animals.” Male discussant, Highland zone*.

*“And this foot and mouth [disease] and skin diseases can be transmitted from one animal to another.” Male discussant, Lowland zone*.

The precautionary measures taken by a few farmers to enhance the health of their animals were deworming, spraying the animals with acaricides or dipping where tick control facilities and services existed. Vaccines were predominantly provided by the veterinary department to control disease outbreaks such as FMD, LSD and CBPP but provision was irregular. Further, not all farmers presented their animals for vaccination.

#### iv. Probability of disease spread through introduction of infected animals in the herds

Focus group discussants reported that at household level, new livestock was acquired through purchase from another producer at farm gate or a stock trader in the livestock market. Similarly, producers sold their animals to other farmers, stock or meat traders at farm gate or the livestock market as demonstrated in the quotes below.

*“Mostly we sell to the slaughter people [people working in slaughter facilities] and butcheries.” Male discussant, Highland zone*.

*“Even me, I can buy from someone here and go and sell during the next auction [livestock market day]. We have livestock brokers here.” Male discussant, Riverine zone*.

Most producers sold their livestock in December, January, February, April and August coinciding with religious festivities, school opening calendars and planting seasons. Animals purchased were generally not isolated from the older herds.

#### v. A regression analysis of good producer practices and socioeconomic characteristics

A regression analysis to determine exposure to risk practices was conducted (Table 2). The regression analysis shows that respondents from the lowland, midland, and riverine zones had less good biosecurity practices than those from the highland zone. By sex, women had more good practices than men. Respondents with primary education had more good practices than those without any formal education. By ethnicity, the pastoral community (Ichamus), had less good practices than the agro-pastoralists (Tugen). Self-employed respondents engaging in service provision had more good biosecurity practices than crop farmers. There were no significant differences in practice based on household type, marital status and age.

**Table 2:** A regression analysis of good producer practices and socioeconomic characteristics.

	Estimate	Standard Error	t value	*p-value*	Significance^a^
(Intercept)	**8.640**	**1.158**	**7.463**	**<0.001**	***
Zone 2—Lowland	**-2.548**	**0.432**	**-5.899**	**<0.001**	***
Zone 3—Midland	**-2.691**	**0.376**	**-7.159**	**<0.001**	***
Zone 4—Riverine	**-3.013**	**0.376**	**-8.018**	**<0.001**	***
Sex 2—Female	**2.381**	**0.296**	**8.040**	**<0.001**	***
Marital status—married monogamous	0.152	0.869	0.175	0.8608	
Marital status—married polygamous	-1.019	0.949	-1.073	0.2836	
Marital status—separated	-0.822	1.268	-0.648	0.5171	
Marital status—divorced	-0.872	1.000	-0.872	0.3837	
Household type—male only no female adult	-0.114	0.660	-0.173	0.8624	
Age in years	0.007	0.010	0.701	0.4834	
Education 2—primary level	**1.034**	**0.410**	**2.523**	**0.0119**	*
Education 3—secondary	0.745	0.506	1.472	0.1416	
Education 4—tertiary	0.568	0.644	0.882	0.3780	
Tribe 2—Ilchamus	**-2.590**	**0.509**	**-5.086**	**<0.001**	***
Tribe 3—Turkana	0.881	2.222	0.396	0.6920	
Tribe 4—Other	-0.141	0.760	-0.185	0.8533	
Main livelihood activity—livestock farming	0.065	0.356	0.183	0.8552	
Main livelihood activity self -employment (service delivery)	**1.618**	**0.522**	**3.101**	**0.0020**	**
Main livelihood activity—self-employment (goods delivery)	-0.217	0.447	-0.486	0.6270	
Main livelihood activity—waged employment	-0.258	0.496	-0.521	0.6028	
Main livelihood activity- salaried employment	0.643	0.575	1.119	0.2635	
Main livelihood activity—no employment	0.791	1.166	0.679	0.4975	
Main livelihood activity—other type of employment	2.018	1.372	1.471	0.1418	

^a^Significance codes: 0 < = ’***’ < 0.001 < ’**’ < 0.01 < ’*’.

Residual standard error: 3.003 on 535 degrees of freedom.

Multiple R-squared: 0.3544, Adjusted R-squared: 0.3255.

F-statistic: 12.24 on 535 and 24 DF, p-value: < 0.001.

### 2. Markets

#### Stock trader characteristics

A total of 203 stock traders were interviewed. Of these 39.4% were from Marigat, 33.5% from Barwessa and 27.1% from Kaptara markets. Each market had a specific trading day assigned to it by the County veterinary department; Kaptara on Tuesdays, Barwessa on Wednesdays and Marigat on Thursdays. Marigat market operated weekly whereas Barwessa and Kaptara operated fortnightly in alternate weeks. Of the 203 traders, 22.7% were sellers, 23.3% buyers and 54.2% both seller and buyer. Traders were predominantly male (96.1%), Christian (98.5%), mainly had primary (42%) or secondary (37.8%) education. They had a mean age of 38.6 years and median age of 37 years with a range 18–68 years.

**Fig 2:**
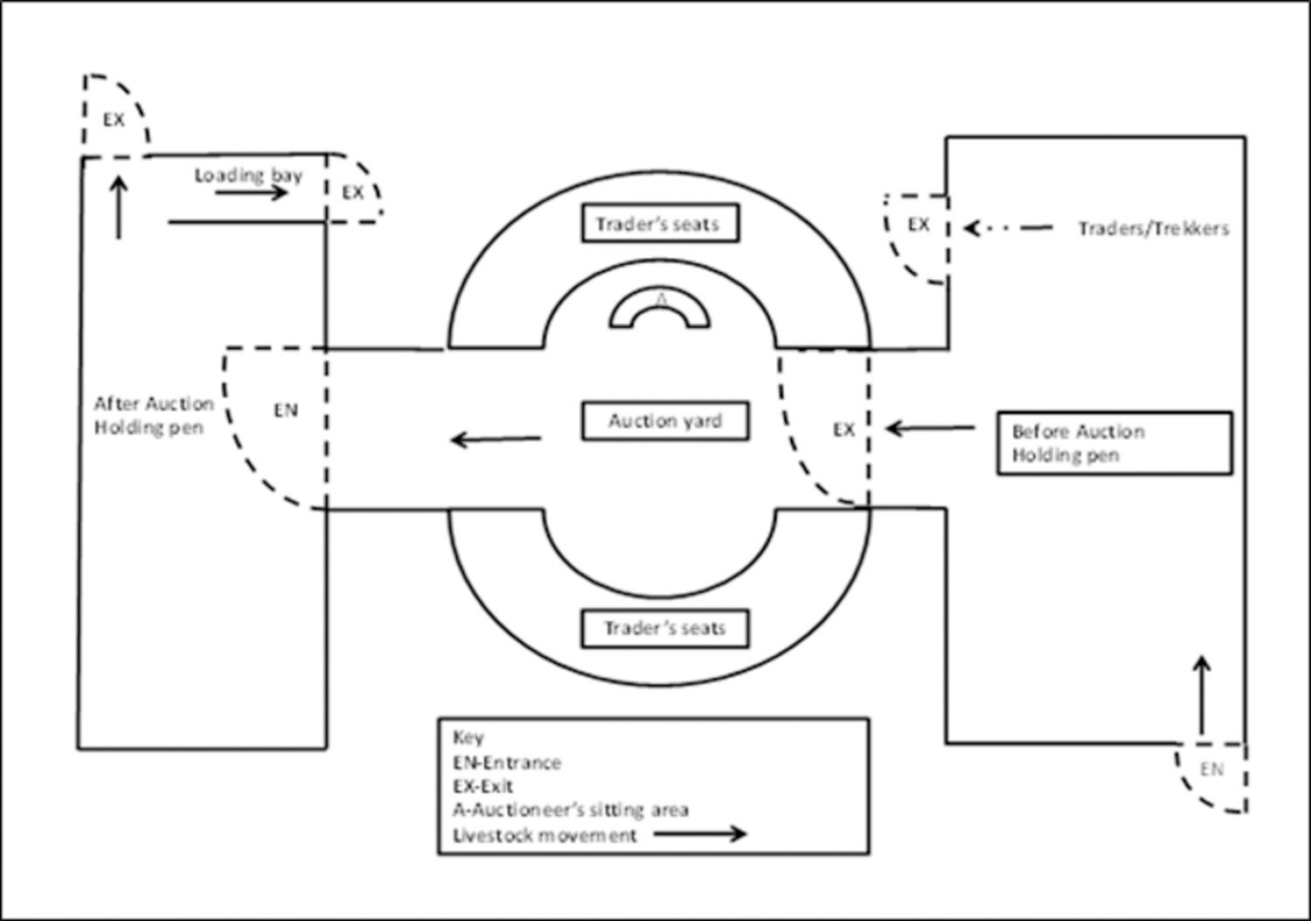
Market layout.

Markets comprised of separate cattle and sheep/goat trading sites. Each trading site was organized into four areas: pre-sale holding pen, auction yard, post-sale holding/loading pen and the isolation zone for sickly animals (Fig 2). All the animals intended for sale were enclosed in the pre-sale holding pen and could only move to the auctioning yard when their time for sale came. After sale, the animals were moved to the post-sale holding area until all due taxes were paid and buyers were issued with livestock movement permits. Livestock were traded in two main ways, through public auction or private negotiations between buyers and sellers. More animals were however sold through auctions run by the county veterinary department. Traders preferred the method for its ability to provide them with fair prices. On the other hand, it enabled the veterinary department to manage livestock trade by collecting taxes from traders, issuing livestock movement permits, controlling sale of sickly animals and effecting trade bans in case of notifiable diseases. Animals traded outside the auction remained in the pre-sale holding pen until requisite permits were issued and taxes paid. Animals that were not sold were released back to their owners when the auction was over.

**Fig 3:**
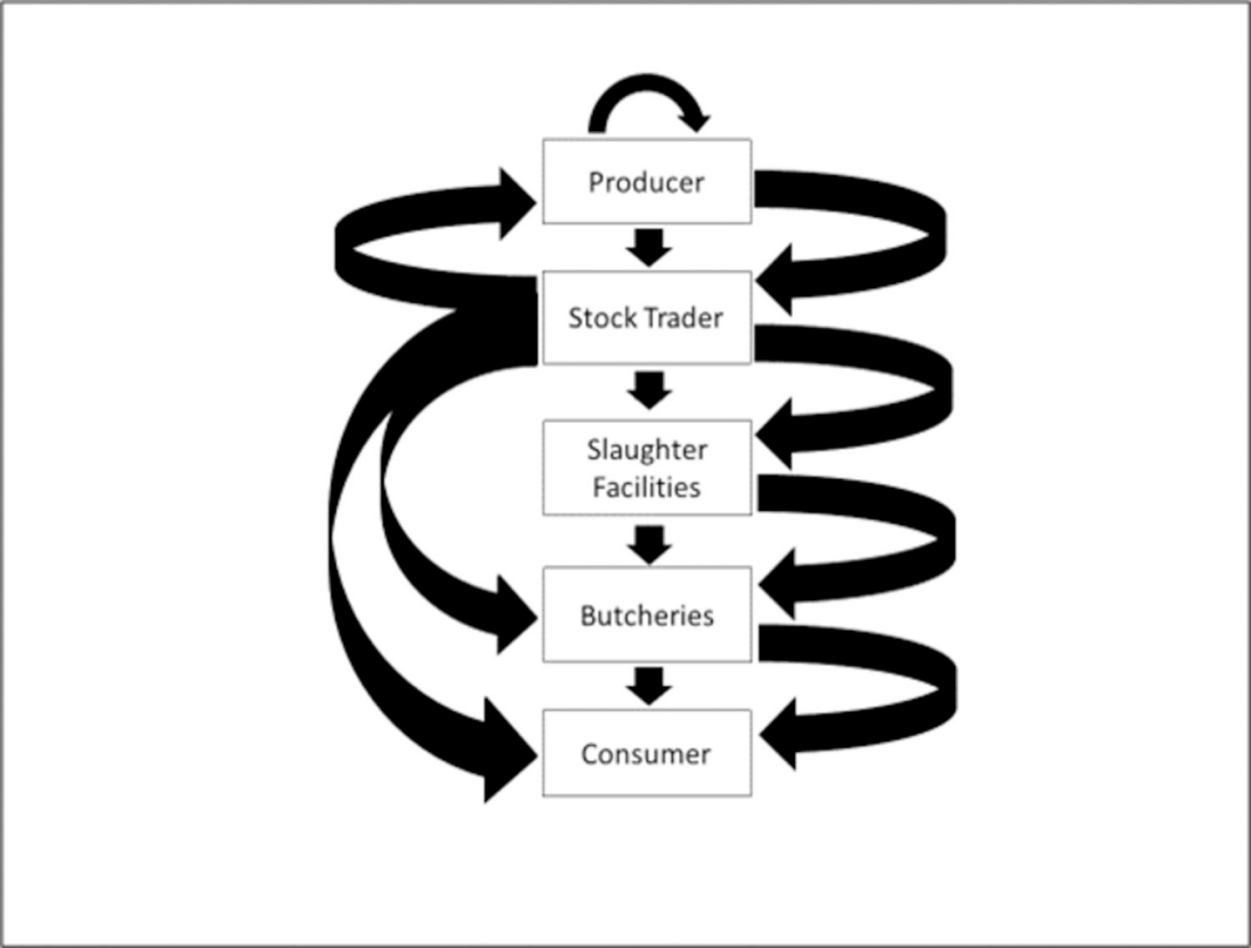
Livestock value chains in the study site.

Sellers (n = 170) in the three markets sourced their stock from East Pokot, West Pokot and Baringo counties from multiple sources which included private farms (32.4%), purchase at farm gate (30%) or purchase and resale in the same market (58.2%). Purchased animals were not only moved to new areas within the County, but were also traded in Nairobi, Nakuru, Uasin Gishu and Elgeyo Marakwet counties. Buyers purchased livestock for trading in meat in butcheries (76.8%) and abattoirs (35.1%), stock (29.8%), farming (7.1%) or domestic consumption (1.2%). Among those who traded in stock, 58.9% resold the animals to traders in other markets, 51.9% in butcheries, 22.2% in abattoirs, 17.1% to farmers and 1.9% for domestic consumption. A single trader could sell their stock to different buyers indicating that the livestock could follow different value chains before reaching the end user (Fig 3).

### Risk in trade

#### i. Probability of an animal being infected with disease during transportation/movement

Most traders (96.2%), took their livestock to the markets on foot. After sale, a higher proportion of livestock were taken to their next destination on foot (60.1%), either by their new owners or hired trekkers. It was not uncommon for trekkers to put together livestock purchased from and by different traders for purposes of walking them to the desired destinations. The next preferred transportation means was vehicles (37.9%). The least used methods of transportation included the use of motorcycles (0.5%) and half foot half vehicles (1%). Among the traders that used vehicles to relocate their livestock (n = 76), some did not attempt to clean the vehicle (26.3%, CI 15.8–38) while others swept it clean (22.4%, CI 11.8–34), laid it with sawdust (18.4%, CI 7.9–30) or washed it with plain water (18.4%, CI 7.9–30). Sawdust was used to make the animals comfortable during transit and as a barrier to animal waste going to the surface of the vehicle. After ferrying livestock, 19.7% (CI 9.2–32) of traders cleaned the vehicle with soap, water and disinfectant, 27.6% (CI 17.1–39.9) with water and soap, 34.2% (CI 23.7–46.5) with plain water, 7.9% (CI 0–12.3–20.2) swept the vehicle and 10.5% (CI 0–22.8) took no action.

#### ii. Probability that an animal dies en route to or from the market

When animals died en route to or from the market, traders disposed them off in different ways. The cadavers were mainly buried (32%, CI 25.6–38.4), skinned and consumed (29.6%, CI 23.6–36.2), burned (14.8% CI 10.3–19.6), or thrown away in the open (10.3%, CI 6.8–14.7). Few traders also had animals skinned before burning (5.4%, CI 3–8.5), thrown away in the open (2%, CI 0.9–4.6) or fed to dogs (2%, CI 0.9–4.6). The skin/hide was used as evidence to the trader that an animal actually died on transit and was not lost by trekkers since most animals were moved on foot.

#### iii. Probability that a sick animal is selected for sale

**Fig 4:**
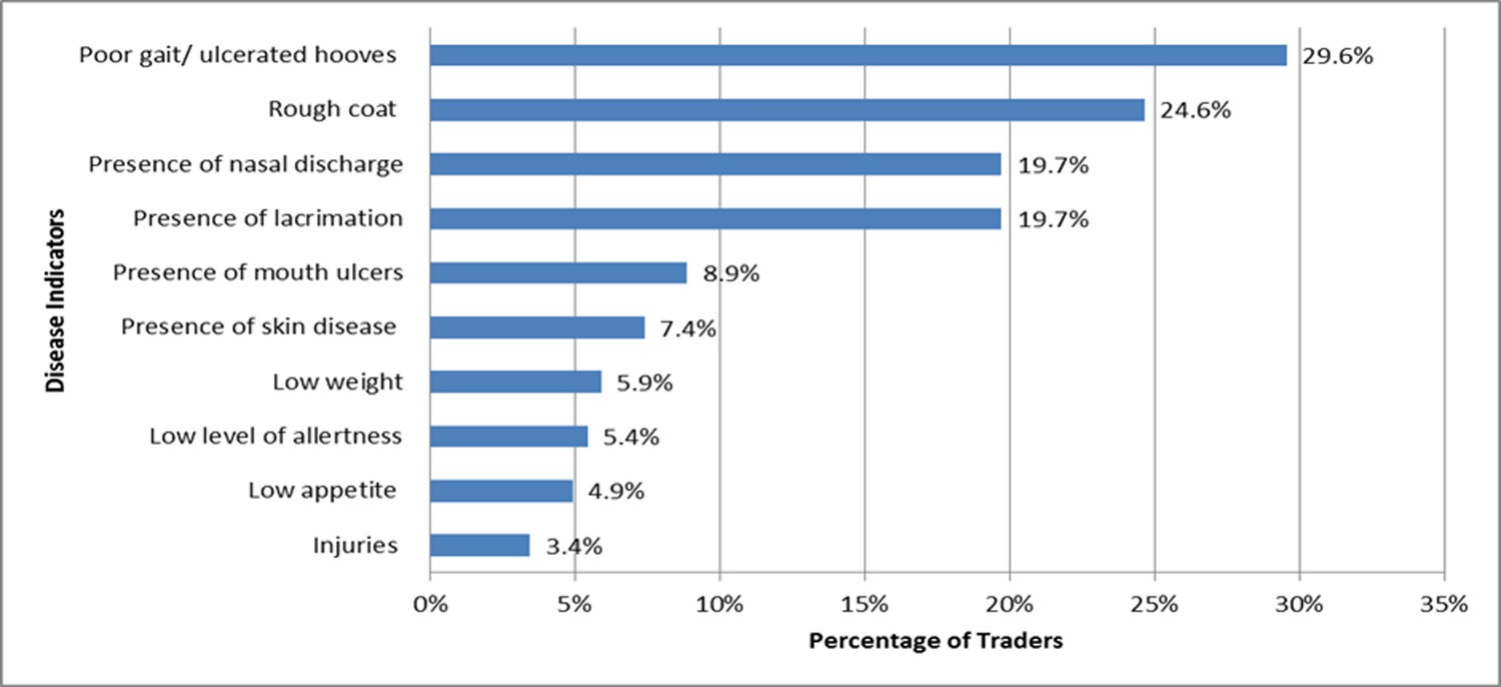
Livestock disease indicators.

Sellers monitored cattle, sheep and goats that were brought to the market for disease before and while on transit. A total of 21 disease signs and symptoms were identified as the criteria used to identify sick or unhealthy animals. The main indicators identified were poor gait/ulcerated hooves, rough skin/hide, presence of nasal discharge, lacrimation or injuries and low weight, appetite and levels of alertness (Fig 4). The others included coughing, weakness, infestation with visible parasites, labored breathing, diarrhea, bloody stool, hard dung, infected udder, frequency of and colour of urination, animal smell, swollen lymph nodes and profuse salivation. Animals brought to the markets did not have any documented health/disease histories.

#### iv. Probability that an infected animal passes pre-market inspection

At the market, 85.2% of traders reported that livestock were further inspected for disease. Slightly more than half of the traders relied on a veterinary officer to inspect the animals, 39.6% inspected for themselves, and 4.4% relied on inspection by both the veterinary officer and themselves. Animals that were found to be sickly were isolated and owners were not allowed to sell them but were instead advised on how to have them treated before returning them to the market for sale. During major disease outbreaks such as RVF, FMD and LSD market closures were imposed by the veterinary department. In these periods, 86.2% of the traders reported ceasing operations in the affected markets. However, 34.5% moved their operations to unaffected markets to keep their businesses afloat and returned when the closures were lifted. This enabled the veterinary department to control disease spread through livestock movement.

### 3. Abattoirs and slaughter slabs

Sampled slaughter facilities operated between 3–7 days processing between 4–84 goats, 1–70 sheep and 1–50 cattle per week though cattle were only slaughtered in the main abattoir. Meats from these slaughter facilities were consumed locally. The facilities had between 1–12 workers, most of whom were male, who had not received any training on good practice in the last 6 months. They were, however, required to seek medical clearance and have valid health certificates every six months to work in the abattoirs/slaughter slabs.

### Risk of worker infection in slaughter slabs and abattoirs

#### i. Probability that an uninspected animal is slaughtered

Animals intended for slaughter in the main slaughter houses were subjected to ante-mortem evaluations and the meat inspected before release to the market. However, this was not always the case in some slaughter slabs where animals were slaughtered as soon as they arrived at the facility. In response to major disease outbreaks, slaughter facilities reported closure whenever slaughter bans were imposed by the veterinary department notably during RVF, FMD, LSD and Anthrax outbreaks.

#### ii. Probability that abattoir workers come into direct contact with infected animal products

**Fig 5:**
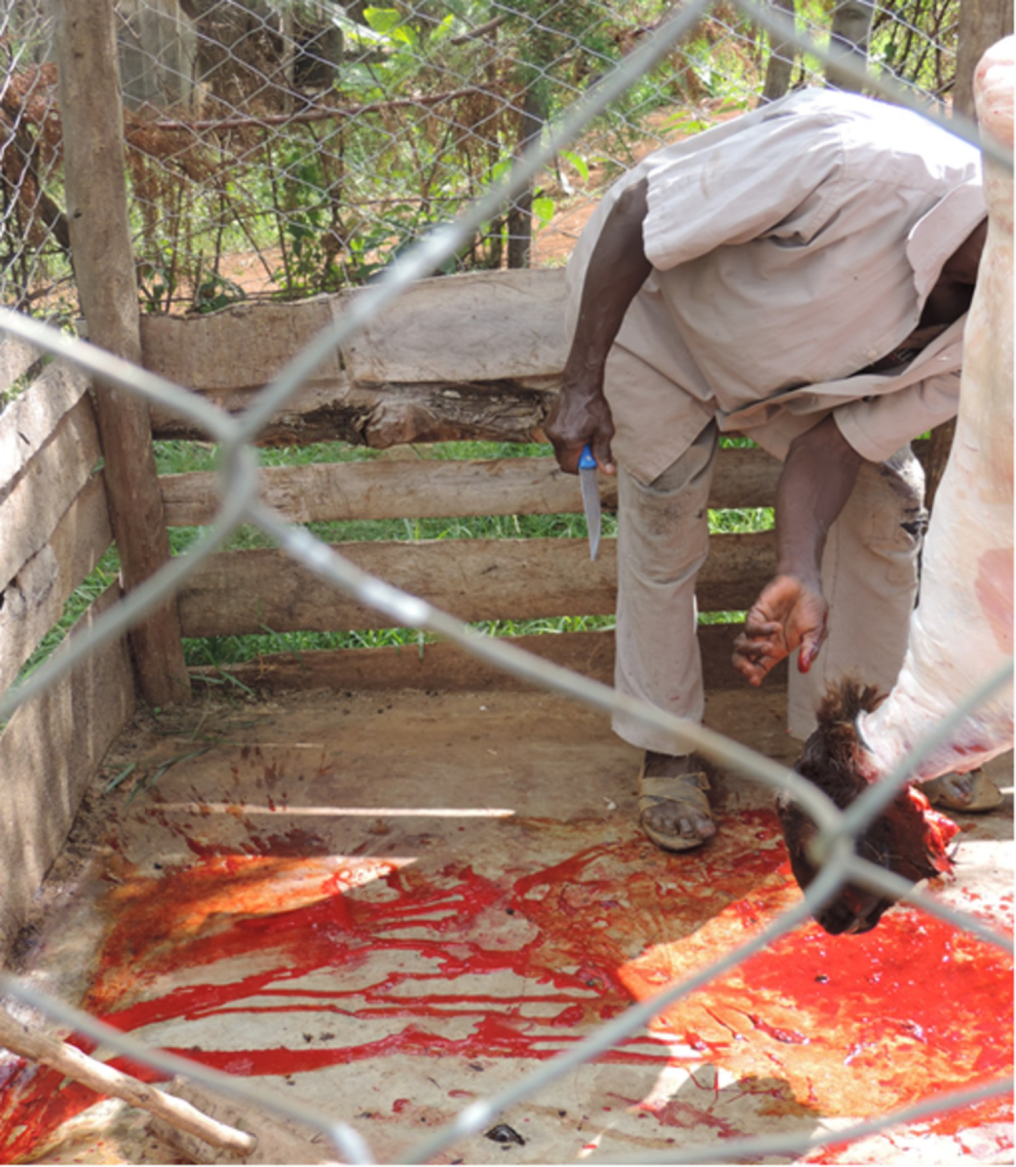
Slaughter slab worker without protective clothing.

All workers in the three main slaughter houses wore white coats/overalls and gum boots while on duty but only a handful had caps. In 3 slaughter slabs, part of the workers wore coats, gumboots or caps but none had the three. None of workers in the remaining four slaughter slabs wore any protective clothing such as gloves or gum boots (Fig 5). In some slaughter facilities, people other than the workers were allowed to assist with slaughtering activities even without protective clothing. Most workers in the slaughter facilities were male, but few women engaged in cleaning offals.

#### iii. Probability that an infection is spread to workers due to poor sanitation

**Fig 6:**
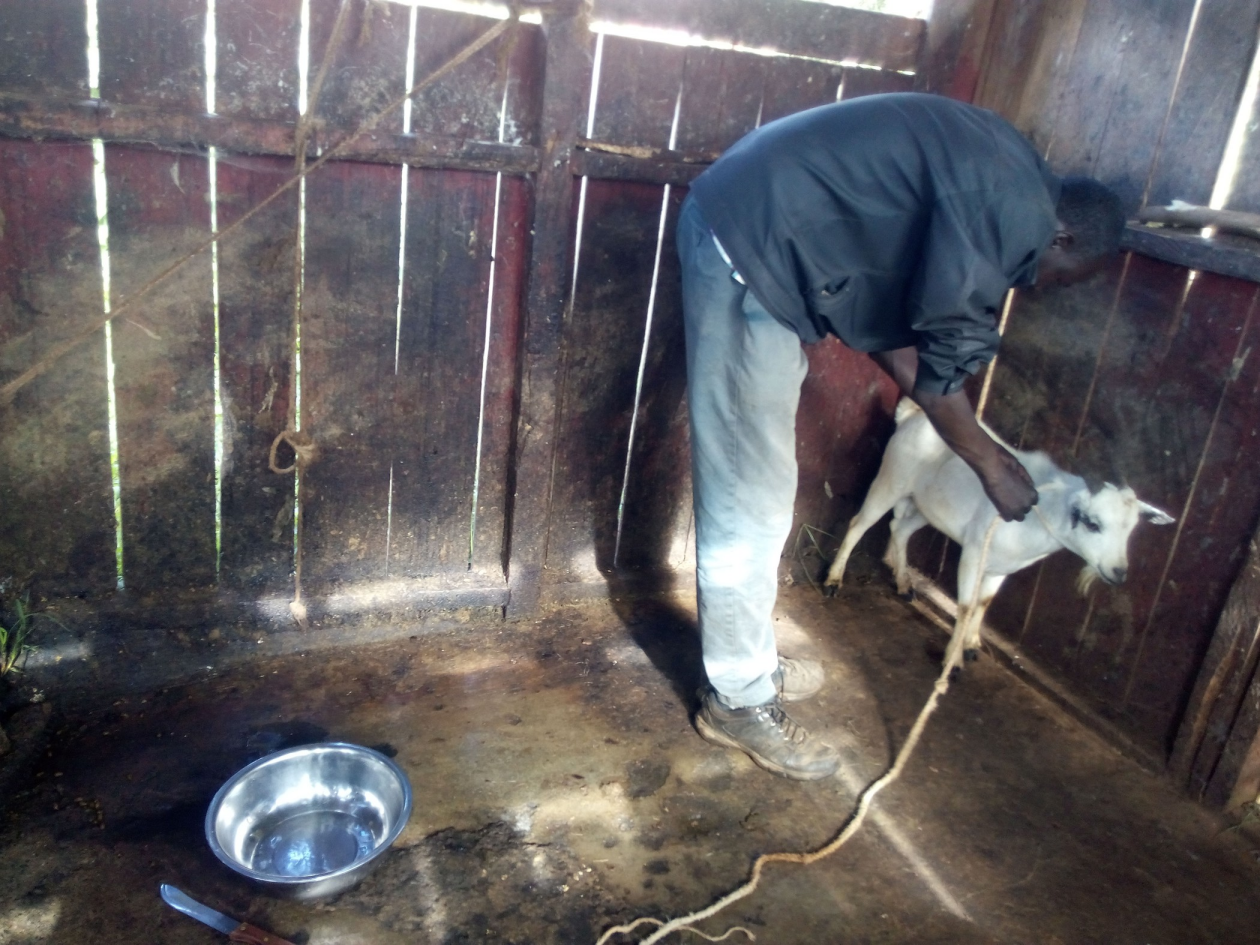
Poor sanitation at a slaughter slab.

**Fig 7:**
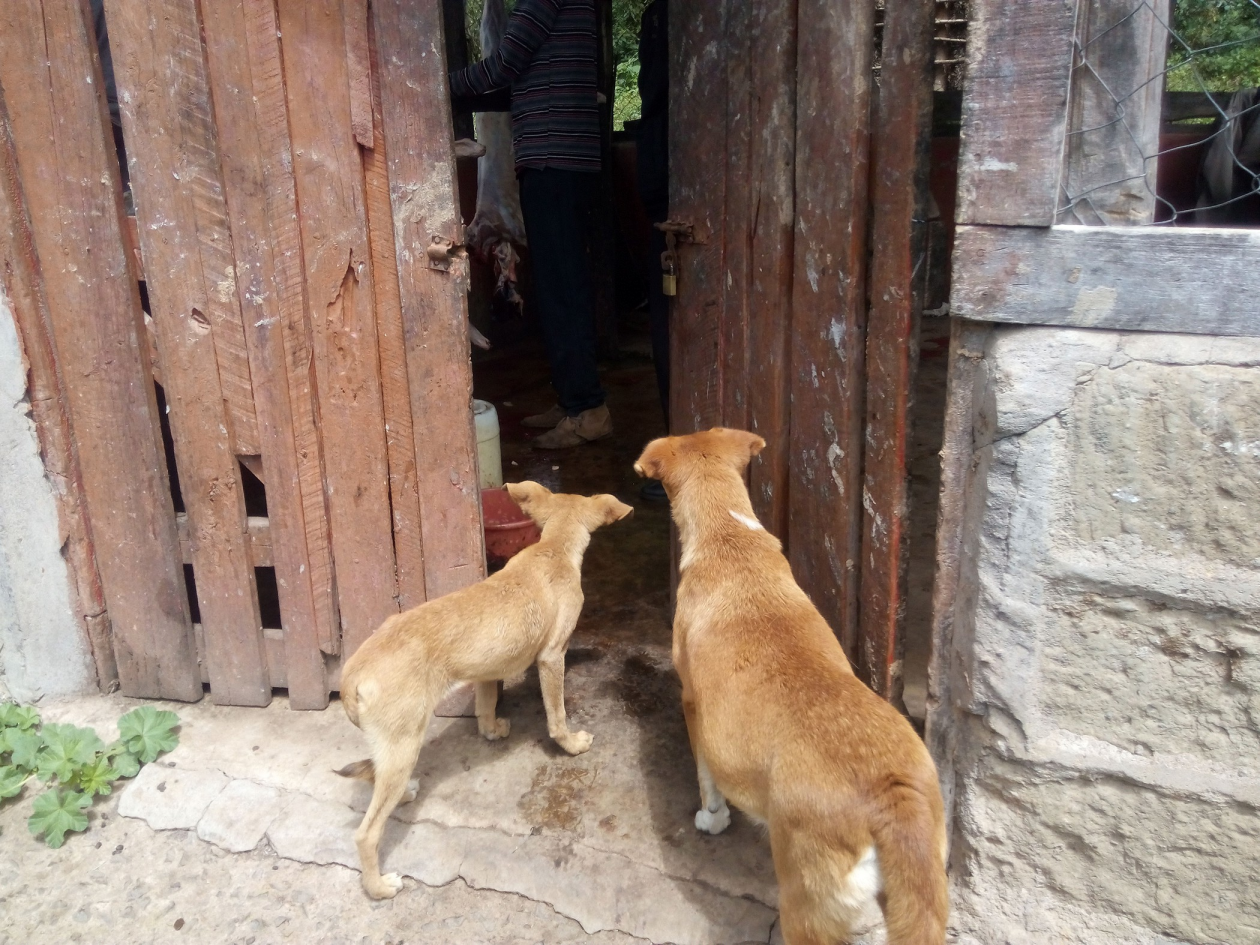
Dogs at the entrance of a slaughter slab.

Of the 10 facilities, 3 slaughter slabs did not have condemnation pits. In some instances, condemned parts and waste pieces of meat were fed to dogs which had easy access to the facilities’ compounds. Only 2 facilities had water piped into the building. However, none had a continuous supply of running water making cleaning difficult in some facilities. Cleaning was done with soap and water only; disinfectant was rarely used. Five slaughter slabs drained their waste water on open ground while the rest had cess pits. In all slaughter houses, manure was mainly discarded in open pits. Figs 6 and 7 illustrate sanitation shortfalls and dog access to slaughter slabs.

## Discussion

Biosecurity measures are important for disease prevention, control and management in the livestock sector. Although in some instances their adoption may be undertaken voluntarily, it may also be a legal requirement enforced by government through food safety experts, public health officers and veterinary health officers as a means of safeguarding human and animal health. This study attempts to assess adoption of biosecurity practices across a cattle, sheep and goat value chains continuum to demonstrate where risks lie. This was achieved by targeting three categories of actors, namely, producers/farmers, traders and slaughter facility workers who were instrumental in ensuring that appropriate measures are undertaken in production, trade and slaughter levels, respectively.

### Producers

At producer level, risks of disease transmission and spread were demonstrated from contact and consumption of milk, meat and blood; handling of sick animals and their products; grazing and watering and introduction of new animals into the herds. Through consumption patterns; contact with infectious animals and animal products; and disposal of dead animals, there lies risk of people contracting zoonotic diseases such as brucellosis, anthrax and RVF which farmers reported to occur in the study area. While brucellosis and anthrax were common occurrences, RVF had occurred only once in the area in 2007.

While seemingly simple biosecurity/food safety measures like milk boiling, consumption of well-cooked meat, and non-consumption of blood from uninspected animals have been advanced, their adoption may not be automatic due to local cultures on food consumption. For example, a study in Ijara, Kenya, found that only 17% of the pastoral communities consumed boiled milk [11]. Among the Maasai community, milk boiling is not favoured because it is assumed to deplete milk of nutrients; change it to a water like consistency; and make it less filling [12]. Other reasons given for preference of raw milk among pastoral communities in East Africa is that boiling makes milk less tasty [13]. As found in this study, the consumption of raw meat and fresh blood has been part and parcel of pastoral diets for many generations [13,14]. Similarly, the use of livestock products as part of treatment processes for sick persons has also been reported in other studies. For instance, among pastoral communities in Ijara, Kenya, who considered blood to be highly nutritious, women who had recently given birth were fed with raw sheep blood as a means of replacing that which they lost in childbirth [15]. In the same region, raw blood, milk and fat derived from sheep were used in home treatment of persons infected with RVF [14,15]. The practice thus may put those involved in milking and slaughtering processes at household level during an RVF outbreak at risk of infection with the disease [16–18]. Brucellosis can also be spread to human beings through consumption of raw milk and blood or handling of aborted materials [13,19–21]. As demonstrated in this study, pastoral communities were more likely to engage in risk practices related to handling and consumption of milk and raw blood than the agro-pastoralists. Since these risk practices may have been practiced previously without any perceived negative effect, calls to change them at community level could encounter resistance for going against food and feeding culture.

Within the study site, meat from home slaughters or skinned dead animals were consumed without being inspected by a trained veterinary officer. It may however be subjected to traditional methods of ensuring safety for consumption such as observation, boiling in herbs, observing ant behavior when exposed to the meat or spleen response when sprinkled with soil, techniques that are not scientifically proven to work [22]. This practice carries the potential for consuming infected meat and transmitting zoonotic diseases such as RVF, anthrax and brucellosis to people. Indeed, during the 2006–2007 RVF outbreak in Kenya, slaughtering, skinning and consumption of infected meat were identified as some of the ways through which people got RVF in Baringo county [23]. Similar observations have been made on human infection with anthrax in Maragua and Garissa areas [24–26] and brucellosis among pastoral communities in Kenya [19].

Disease management was reported to be largely in the hands of farmers as they preferred to treat their animals for themselves without involving veterinary officers due to limited access to veterinary professionals and/or the costs associated with seeking professional veterinary services [22]. This practice is especially dangerous because farmers may be self-treating disease symptoms, for instance abortion in their animals, and may get infected if dealing with a zoonotic disease like RVF and brucellosis. Moreover, that there was consumption of milk from sick animals which were probably being self-treated with antibiotics, this could lead to antimicrobial resistance in humans from consumption of antibiotic resistant bacteria in contaminated milk or antibiotic residues [27].

The surest way to prevent zoonotic infections that were reported in the study site such as anthrax, brucellosis and RVF in humans would be to vaccinate livestock and hence prevent the occurrence of the diseases at all [19,24,28]. However, provision of vaccines against these diseases to farmers in Kenya is irregular and when conducted it is erratic often in response to disease outbreaks [19,22–24,29,30]. For example, according to Jost et al. [31], livestock vaccinations against RVF during the 2006–2007 outbreak in Kenya began nearly the same time with the index human case. Even when mass vaccinations are conducted by veterinary departments, uptake can be hampered by barriers such as cost, difficulties in taking livestock to vaccination points and lengthy distances to vaccination sites [28,30].

Proper disposal of infected animals or animal parts through burying or burning as advocated for by the veterinary departments is crucial in disrupting disease transmission cycles, especially for zoonotic diseases like anthrax [24], cerebral coenurosis [32], RVF [22,23] and brucellosis [19,26] that were reported to occur in the study site. In the case of anthrax, deep burying of infected animals reduces the risk of introducing the causative agent, *Bacillus anthracis* spores, to soil and vegetation from where they are ingested or inhaled by animals while grazing which can cause disease in them and set grounds for human infection [24]. Besides contact of the infected animal or animal products with the ground, dogs, birds and insects can spread spores to the environment [24,25]. Cerebral coenurosis, which is one of the diseases reported to occur in sheep and goats is caused by Coenurus cerebralis, the larval stage of the tapeworm *Taenia multiceps* which lodges in the brain [33,34]. If the brain of an infected goat/sheep is fed to dogs as a means of disposal, upon defecation they will introduce the eggs to the soil which can be ingested by other goats and sheep while feeding thus maintaining the disease cycle [32–34]. For RVF and brucellosis, which cause abortions in infected animals, handling of the abortus or birthing fluids with bare hands in the process of caregiving or disposal can lead to human infections [19,22,26].

In the study site, in addition to burning and burying, farmers considered consumption as a means of disposal. Burying and burning may have been less popular among farmers due to the prevailing beliefs on what constitutes proper ways of disposing an animal from a cultural perspective [22]. For example, according to the Ilchamus from the lowland zone, interment was only considered a preserve for human bodies and not livestock [22]. Other risky beliefs held in other parts of Kenya were that dead animals should be opened up before disposal [24] and that if animals were buried without being skinned it would cause death in the remaining living animals [22,30]. These examples demonstrate the contestations that exist between need for food, loss mitigation and observing cultural norms and biosecurity practices demanded by food safety and veterinary health experts.

Livestock movement [35], embedded in nomadic movements, communal grazing and watering increases the risk of animal exposure to diseases, pathogens and pests [19,36]. In such farming systems, other biosecurity measures to cover the risks presented are needed. For example, regular spraying of cattle with acaricides will keep off pests such as ticks which have been associated with diseases like East Coast fever although an alternative would be to have the animals vaccinated against the disease which may not be affordable to all farmers [37]. In prevention of trypanosomiasis, techniques such as aerial and ground spraying; introduction of sterile male tsetse flies to curtail multiplication; trapping; and treating cattle with insecticides to keep off tsetse flies can be used [38]. Isolation of new animals before mixing them with existing herds can prevent introduction of disease to clean animals [26]. Regular deworming of small ruminants and dogs with anthelmintics can aid in breaking cerebral coenurosis’ disease cycle [33,34].

### Stock traders

At the trade level, the veterinary department, commercial and non-commercial traders were keen to ensure that the livestock they traded in were healthy, albeit based on physical appearance. The knowledge of disease signs and symptoms to look out for indicated that they had their own criterion for determining the qualities that a healthy animal should have and identifying animals with ill health. This was useful in ensuring that traders got value for money and averting losses from buying sickly animals which may die thereafter or require additional investment to bring them to good health. It is recommended that animals (cattle) be isolated for two weeks before being introduced to herds, but this was not observed [39].

The main livestock movement strategies used—trekking and vehicles–carried the risk of bringing together healthy and ill/infested animals, a route through which diseases and pests can be spread from one animal to another. Crowding during transportation could also cause stress in animals which can induce disease. That less than 20% of traders cleaned the vehicles with water, soap and disinfectant before or after ferry livestock may indicate a lack of awareness that some livestock diseases pathogens can be spread from surfaces. Moreover, since most traded livestock were intended for trading as meat in butcheries and slaughter facilities or resale as stock, the traders may also have seen no value in over-investing in the wellbeing of animals that would soon leave their hands and cease to be their responsibility.

The disposal strategies of animals that died to and from the market differed from producers in that more traders reported burying animals that died, followed by consumption and burning. The proportion reporting burying and burning as the practiced disposal techniques may have done so because as traders, they were aware of what the veterinary department expects of them but not necessarily that they observed the practice. It is also a possibility that the proportion that reported skinning and consuming dead animals was lower in proportion than is the case in actual practice because of trader desire to maximize of profits. A study in Kenya exemplified this by reporting that people have been infected with anthrax from the practice of selling meat from dead animals cheaply as a means of loss reduction [24]. This may be fueled by anthrax disease in livestock having no ante-mortem symptoms and is only noticeable upon sudden death in animals [24,30]. In pastoral communities in Ngorongoro, Tanzania, sheep and goats infected with cerebral coenurosis were sold for slaughter [34], just as was reported in this study. While their meat may not have a negative effect on humans, if the infested brain is fed to dogs it could sustain the disease cycle.

Quarantines and market, slaughter facility and butchery closures effected by the veterinary departments when outbreaks of notifiable diseases like FMD, LSD, RVF occurred was protective for the traders, farmers and livestock in the County. Quarantines limit animal movement and consequently reduces the risk of introducing disease where it has not spread to already [40], as other measures are taken to contain it in the outbreak zones. They also present an opportunity for making traders and farmers aware of the disease risks they are faced with at the time hence increasing their knowledge in matters of livestock health. However, a negative effect of livestock and livestock product trade ban was the temporary loss of means of earning livelihoods among affected traders and farmers.

### Slaughter facilities

In the abattoirs, there were concerted efforts in ensuring that all animals presented for slaughter were free from visible signs of disease and all meat was certified safe for consumption by veterinary personnel before sale. However, this was not always the case in slaughter slabs indicating the risk of slaughtering and selling a sick animal’s meat to an unsuspecting public. This was contrary to the Meat Act [4], which dictates that animals should only be slaughtered after they have gone through an ante-mortem and carcasses cleared for sale by an inspecting veterinary officer. The practice of the veterinary department invoking and imposing bans on slaughter and trade of animals and meats for instance during RVF outbreaks was protective to slaughter facility workers and the local populations. Although it decreased the risk of disease transmission by contact with infected animals and consumption of infected animal products, it led to temporary loss of jobs and incomes for the workers that operated within the provided regulations.

It is a requirement that slaughter facility workers wear clean head coverings, bright coloured coats/overalls and gumboots as protective clothing while on duty [4]. It was however observed that this regulation was not adhered to in all slaughter facilities implying that the regulation was not enforced as required. Similar practice has been observed elsewhere in Kenya [41]. Non-use of recommended protective clothing exposes workers to the risk of coming to direct contact with infectious materials and spreading them from their workstations to other public and private spaces. The same risk would also apply to members of the public accessing slaughter areas.

As a sanitation requirement by the Meat Act [4], ample portable water supply is needed but most of the sampled facilities did not have that. Baringo County being an arid area, only small sections have access to piped water. Majority of users source water from streams, rivers and or lakes. Water shortages can compromise cleanliness levels of slaughter facilities, equipment, workers and the meat products prepared therein due to the nature and volumes of waste generated in the slaughtering process. The requirement that slaughter equipment should be cleaned in hot water, at 82 degrees Celsius [4], was not observed in any of the facilities, a challenge possibly presented by lack of requisite heating infrastructure. The non-use of disinfectant in cleaning slaughter facilities implied that although a reasonable level of cleanliness may have been observed, there was risk of contamination from floors, surfaces and equipment harbouring disease pathogens that cannot be denatured by soap and cold water only. Proper disposal of condemned parts and carcass debris was observed to be limited in slaughter slabs lacking condemnation pits. This encouraged disposal in open spaces, giving rats/mice, dogs and birds access which contravenes the Meat Act [4]. Accumulation of manure within slaughter facility compounds is discouraged as it can lead to effluence discharge, consequently contaminating crops and water in their environs [36].

This study is not without limitations. Except for the observation data collected in slaughter facilities, all other data was self-reported by producers and traders. There is thus a risk of participants having overstated the biosecurity practices known to be right and understated those known to be improper. Biosecurity being a broad subject, the researchers picked on a few elements relevant to the study site. The findings of this study can therefore be strengthened by further studies to generate information on the missing dimensions such as the symptoms of disease slaughter facility workers look out for in the animals they slaughter; the sale of uninspected meats from animals slaughtered outside slaughter facilities; and how to cultivate trust and cooperation among veterinary departments, producers, livestock traders, and slaughter facility workers.

## Conclusion

This study has demonstrated that implementation of biosecurity measures is not as straight forward a process as may be envisioned in biosecurity frameworks and government policy documents. By focusing on three cattle, sheep and goats value chain actors namely producers, traders and slaughter facility workers, it has shown the biosecurity risks that exist at each level and how they occur in practice. This exemplifies how a One Health approach to biosecurity in local settings can be advantageous to animal, human and environmental wellbeing by pointing out the interconnectedness of each and how an action by one value chain actor or sector affects the health outcomes of another. For example, proper disposal of animals with anthrax can limit contamination of the environment with disease spores which can be ingested by animals leading to disease and risk of human infections.

Due to low education levels across zones and higher vulnerability in men than women, the study has demonstrated the importance of having agricultural extension services in rural settings, through which trainings and practical examples can be provided to the various actors to make them aware of the biosecurity risks that can occur and how to mitigate them. While awareness creation does not always guarantee uptake and behavior change [42], it is critical in creating and expanding understanding of the circumstances which actors find themselves in and the associated biosecurity risks. Opportunities for knowledge transfer that can be utilized to increase awareness on biosecurity measures include home visits for farmers; short talks in the market as the veterinary officers prepare to begin livestock trade; and in the process of conducting inspections of slaughter facilities and meat produced therein. However, for these to occur, there is need for adequate numbers of veterinary/agricultural officers to be recruited and provided with the necessary facilitative resources such as motor cycles and fuel for transport; veterinary medicines and consumables such as syringes and gloves for livestock treatment and disinfectant for slaughter facilities; and structures that allow for farmers trainings.

The study has shown that there can be contestations between observing cultural practices and biosecurity requirements. Actor willingness to adopt biosecurity measures is linked to the perceived benefits of such actions [41]. Where gain is perceived to be minimal or non-existent, adoption may be lower [41]. This can lead to limited uptake of measures causing major public health problems [43]. This highlights the fact that top-down biosecurity interventions may not be efficient especially in cases where people do not understand their usefulness or see direct benefit [36]. Moreover, it calls for adapting biosecurity guidelines to local contexts as a “one size fits all approach” may not work in low income countries [44]. However, this should not compromise disease control, management and prevention outcomes envisioned by biosecurity guidelines and policies. This study thus proposes further community focused studies on willingness to adopt biosecurity practices and possible critical points of intervention at county and national levels. The data used in this paper is available as S1–S3 Files.

## Supporting information

S1 File (XLS)

S2 File (XLSX)

S3 File (XLSX)
